# In Utero Exposure to Bisphenol a Promotes Mammary Tumor Risk in MMTV-Erbb2 Transgenic Mice Through the Induction of ER-erbB2 Crosstalk

**DOI:** 10.3390/ijms21093095

**Published:** 2020-04-28

**Authors:** Zhikun Ma, Amanda B. Parris, Erin W. Howard, Meghan Davis, Xia Cao, Courtney Woods, Xiaohe Yang

**Affiliations:** 1 Julius L. Chambers Biomedical/Biotechnology Research Institute, Department of Biological and Biomedical Sciences, North Carolina Central University, Kannapolis, NC 28081, USA; zma@nccu.edu (Z.M.); ablack18@nccu.edu (A.B.P.); ewhoward12@catawba.edu (E.W.H.); xcao168@gmail.com (X.C.); cwoods21@eagles.nccu.edu (C.W.); 2 Biotechnology, Rowan-Cabarrus Community College, Kannapolis, NC 28081, USA; meghan.davis@rccc.edu; 3 The Lineberger Comprehensive Cancer Center, University of North Carolina at Chapel Hill, Chapel Hill, NC 27599, USA

**Keywords:** Bisphenol A (BPA), in utero exposure, estrogen receptor, erbB2, breast cancer, mammary development

## Abstract

Bisphenol A (BPA) is the most common environmental endocrine disrupting chemical. Studies suggest a link between perinatal BPA exposure and increased breast cancer risk, but the underlying mechanisms remain unclear. This study aims to investigate the effects of in utero BPA exposure on mammary tumorigenesis in MMTV-erbB2 transgenic mice. Pregnant mice were subcutaneously injected with BPA (0, 50, 500 ng/kg and 250 µg/kg BW) daily between gestational days 11–19. Female offspring were examined for mammary tumorigenesis, puberty onset, mammary morphogenesis, and signaling in ER and erbB2 pathways. In utero exposure to low dose BPA (500 ng/kg) induced mammary tumorigenesis, earlier puberty onset, increased terminal end buds, and prolonged estrus phase, which was accompanied by proliferative mammary morphogenesis. CD24/49f-based FACS analysis showed that in utero exposure to 500 ng/kg BPA induced expansion of luminal and basal/myoepithelial cell subpopulations at PND 35. Molecular analysis of mammary tissues at PND 70 showed that in utero exposure to low doses of BPA induced upregulation of ERα, *p*-ERα, cyclin D1, and c-myc, concurrent activation of erbB2, EGFR, erbB-3, Erk1/2, and Akt, and upregulation of growth factors/ligands. Our results demonstrate that in utero exposure to low dose BPA promotes mammary tumorigenesis in MMTV-erbB2 mice through induction of ER-erbB2 crosstalk and mammary epithelial reprogramming, which advance our understanding of the mechanism associated with in utero exposure to BPA-induced breast cancer risk. The studies also support using MMTV-erbB2 mouse model for relevant studies.

## 1. Introduction

Environmental exposure to endocrine disrupting compounds (EDCs) plays a critical role in breast cancer etiology. In particular, recent studies suggest that the in utero stage is vulnerable to environmental factors and in utero exposure to EDCs may significantly modify breast cancer risk later in life [1,2,3]. A well-known example is maternal exposure to diethylstilbestrol (DES)-associated breast cancer risk [4]. It is of great significance to identify prenatal environmental factors that alter breast cancer risk.

Bisphenol A (BPA), the building block of polycarbonate plastics, is the most common environmental EDC that binds estrogen receptor alpha (ERα), ERβ, and estrogen-related receptor-γ (ERRγ) [5,6,7]. BPA upregulates a number of ER target genes [8], and may also induce cellular responses through non-genomic pathways by interacting with membrane ER and the G-protein-coupled receptor 30 (GPR30) [9,10]. As one of the most common environmental compounds exposed in daily life [5,6], BPA has been detected in the urine of approximately 95% of the population in the United States [11]. BPA exposure has been associated with increased risk for reproductive abnormalities, obesity, neurobehavioral problems, diabetes, and cancers [12,13,14,15,16,17,18]. In particular, BPA exposure-associated breast cancer risk is a major health concern [19,20,21].

A few previous studies suggest that maternal exposure to BPA may also have a profound impact on breast cancer risk of the offspring [21,22,23]. Results from a Wistar rat model indicated that prenatal exposure to 25 µg/kg bodyweight (BW) BPA via osmotic pumps induced proliferative mammary glands and preneoplastic lesions after a subcarcinogenic dose of *N*-nitroso-*N*-methylurea (NMU) [24]. Prenatal exposure to 25 or 250 µg/kg BW BPA via osmotic pumps in CD-1 mice also induced proliferative mammary glands and hormonal disruption [25]. Another study showed that pregnant Sprague-Dawley rats were exposed to 25 or 250 µg/kg BW BPA via oral gavage during gestational day (GD) 10–21. Female offspring were more susceptible to dimethylbenz(a)anthracene (DMBA)-induced mammary tumorigenesis, which involved upregulation of progesterone receptor (PR)-A, steroid receptor coactivators 1-3, and EGFR/erbB2 in mammary tissues at PND 100 [26]. Results from both rat and mouse models revealed that prenatal exposure to BPA induced early onset of puberty and significant morphological alternations in the mammary glands, including increased terminal end buds (TEBs), ductal growth, and lateral branching at puberty, which were associated with increased proliferation and decreased apoptosis of the epithelial cells [23,24,27,28,29]. Acevedo et al. reported that exposure to environmentally relevant levels of BPA during gestation and lactation induced mammary gland neoplasms in the absence of any additional carcinogenic treatment, and therefore proposed that BPA may act as a complete mammary gland carcinogen [30]. Taken together, the above studies provided proof of concept of in utero exposure to BPA-associated mammary tumor risk and described major phenotypes resulting from the exposure [31,32]. However, the gap of our understanding between in utero exposure to BPA-induced proestrogenic effects and the altered morphogenesis/tumor risk remains significant. Therefore, it is of pivotal importance to understand the underlying cellular and molecular mechanism to facilitate the development of preventative and management strategies. Nevertheless, studies using carcinogen models, are compromised for mechanistic studies because of the significance between carcinogen induced mammary tumors and human breast cancer carcinogenesis. Therefore, we investigated in utero exposure to BPA-induced tumor risk and morphogenic alterations focusing on cellular and molecular mechanisms using MMTV-erbB2 transgenic mouse model in this report.

erbB2 (Her2/Neu) is a proto-oncogene that is amplified/overexpressed in approximately 30% of breast cancer cases [33]. As a typical receptor tyrosine kinase (RTK), activation of erbB2 induces signal transduction in a number of pathways, including PI3K/Akt, MAPK/Erk, and mTOR signaling cascades, involved in tumor cell proliferation and survival [34,35]. Although erbB2 overexpression alone is carcinogenic, erbB2-mediated carcinogenesis can be influenced by environmental factors, such as xeno-estrogenic compounds [36]. MMTV-erbB2 transgenic mice, a well-established model for mammary tumor risk studies, have been used extensively to study breast cancer risk modulated by hormonal and dietary factors, such as tamoxifen, phytoestrogens, and other estrogen modulators [37,38,39,40]. The well-defined pathogenic background of this model is of advantage for mechanistic studies. Therefore, we investigated the effect of in utero exposure to BPA on mammary development and tumor risk of MMTV-erbB2 transgenic mice to provide mechanistic insight into in utero BPA exposure-associated breast cancer risk, which will further our understanding on gene-environment interactions in the tumorigenic process.

In this report, we studied the effects of in utero exposure to various doses of BPA on mammary development and tumorigenesis in MMTV-erbB2 transgenic mice, and characterized molecular signaling in the ER and RTK pathways. The results underscore the significant impact of in utero exposure to BPA on mammary tumor risk later in life.

## 2. Results

### 2.1. In Utero Exposure to Low Doses of BPA Promotes Mammary Tumor Development in MMTV-erbB2 Mice

**Figure 1:**
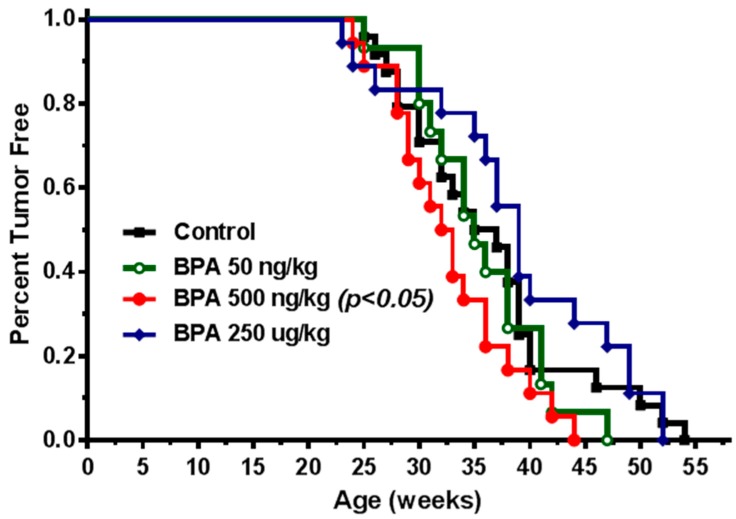
In utero exposure to low dose Bisphenol A (BPA) promotes mammary tumor development in MMTV-erbB2 transgenic mice. Kaplan–Meier tumor free survival curves were calculated based on tumor latency of the MMTV-erbB2 transgenic mice (*n* = 20 mice per group) with in utero exposure to 0 (square), 50 ng/kg (circle), 500 ng/kg (dot), or 250 µg/kg (diamond) bodyweight of BPA daily between GD 11 and 19.

To investigate in utero exposure to BPA-mediated mammary tumorigenesis, we studied the effects of in utero exposure to BPA on mammary tumor risk and molecular signaling using the MMTV-erbB2 transgenic mice. In our preliminary study, the pregnant mice were treated with BPA at doses of 0, 50, and 250 µg/kg BW via subcutaneous injection. Although these doses were chosen based on previous rodent studies using carcinogen models [26], we found that BPA exposure at the given conditions did not promote mammary tumor development of the offspring (data not shown), whereas mammary morphogenesis of the mice in the 250 µg/kg group were altered evidently (detailed below). Considering inappropriate doses were the cause of the above insignificant results, we redesigned the experiments with adjusted exposure doses based on subcutaneous injection. To this end, pregnant MMTV-erbB2 transgenic mice were treated with 0, 50, 500 ng/kg and 250 µg/kg BW of BPA. The group of 250 µg/kg was included as high dose control and for further characterization based on the preliminary study. Tumorigenic analysis of the female offspring indicates that mice from the control group developed tumors between 25 and 55 weeks of age with an average latency of 37.6 weeks (Figure 1). In contrast, mice with in utero exposure to low doses of BPA developed tumors at an earlier age with an average latency of 35.1 and 32.3 weeks for the 50 ng/kg and 500 ng/kg groups, respectively. Statistical analysis indicated that in utero exposure to 500 ng/kg of BPA significantly promoted early tumor development (*p* < 0.05), although tumor promotion in the 50 ng/kg BPA group was not statistically significant. The tumor development in the high dose BPA (250 µg/kg) group did not promote but somewhat delayed tumor development, although it was statistically insignificant. Data from this experiment suggest that in utero exposure to low dose BPA, around 500 ng/kg, has more adverse effects on mammary tumor development.

### 2.2. In Utero BPA Exposure Alters Vaginal Opening Dates and Estrous Cycle in MMTV-erbB2 Transgenic Mice

Since in utero treatments usually affect puberty physiology [25,41], we examined vaginal opening and estrous cycles of MMTV-erbB2 offspring with in utero exposure to BPA. As shown in Table 1, the vaginal opening time in mice with in utero exposure to low doses of BPA (50 and 500 ng/kg) was significantly earlier than that of the control group, indicating an earlier onset of puberty. Interestingly, in utero exposure to the high dose of BPA (250 µg/kg) did not result in a significant change in the vaginal opening dates from the control group.

We next examined the effects of in utero BPA exposure on the estrous cycles of MMTV-erbB2 mice based on stained vaginal smears. Mice from the control group had regular estrous cycles with distinct phases (proestrus, estrus, metestrus, and diestrus). However, the estrous cycle phases of the mice with in utero exposure to low doses of BPA, but not the high dose group, were disrupted. A notable characteristic of the low dose groups was that the number of days in the estrus phase during the observation period was significantly longer than that of the control group (Table 2), indicating systemic hormonal deregulation. These results altogether indicate that low dose in utero BPA exposure induces pro-estrogenic effects that lead to an earlier puberty onset and disrupted estrous cycles with prolonged estrus phase.

### 2.3. In Utero Exposure to BPA Induces Proliferative Mammary Morphogenesis

**Figure 2:**
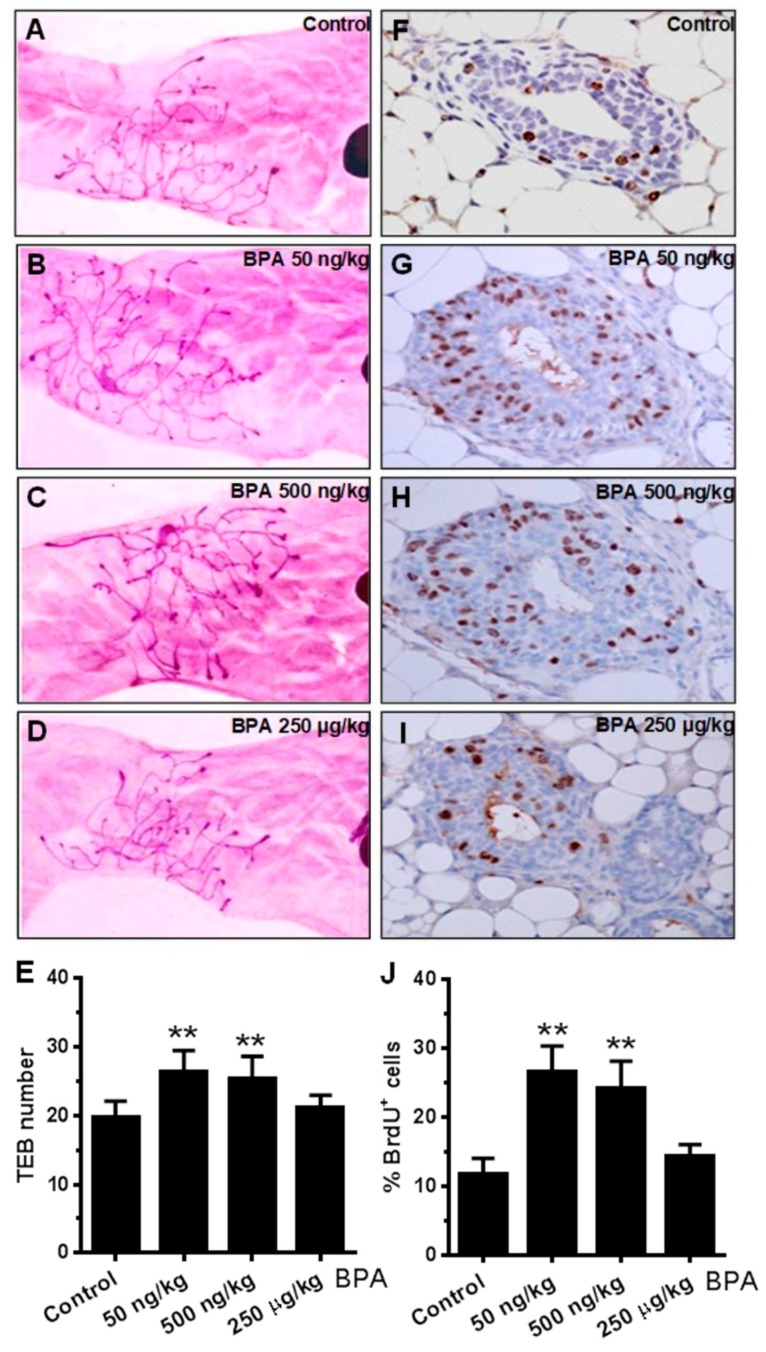
In utero BPA exposure affects terminal end bud (TEB) number and BrdU expression in mammary glands. Representative images are shown of mammary gland morphology and proliferation status. Mammary glands at PND 35 were collected from mice with in utero exposure to 0 (**A**,**F**), 50 ng/kg (**B**,**G**), 500 ng/kg (**C**,**H**), or 250 µg/kg (**D**,**I**) bodyweight of BPA daily between GD 11 and 19. Whole mounts (**A**–**D**) and BrdU incorporation detected with IHC (**F**–**I**) were analyzed. Quantification of terminal end bud (TEB) number (**E**) and BrdU incorporation (**J**) are displayed (** *p* < 0.01).

Advanced mammary gland development and increased ductal/epithelial density are linked to breast cancer risk [42,43]. To understand the extent of in utero BPA exposure on pubertal mammary development, we examined the terminal end bud (TEB) number in mammary glands at PND 35. TEBs are enriched with mammary stem cells that determine mammary development programming, which is sensitive to developmental factors [44]. Figure 2A–E shows that low dose (50 and 500 ng/kg) in utero BPA exposure significantly increased TEB numbers, while the high dose (250 µg/kg) of BPA did not, as compared to the control. Next, we determined the proliferation status of mammary epithelial cells at this stage with BrdU incorporation, a direct indicator of cell proliferation. The results showed that BrdU positive epithelial cells in the glands with in utero exposure to 50 and 500 ng/kg BPA, but not the high dose group, were significantly more than that of the control group (Figure 2F–J). Our data suggest that in utero exposure to BPA at low concentrations induces an increase in TEB numbers and advanced mammary development during puberty in mice at PND 35.

**Figure 3:**
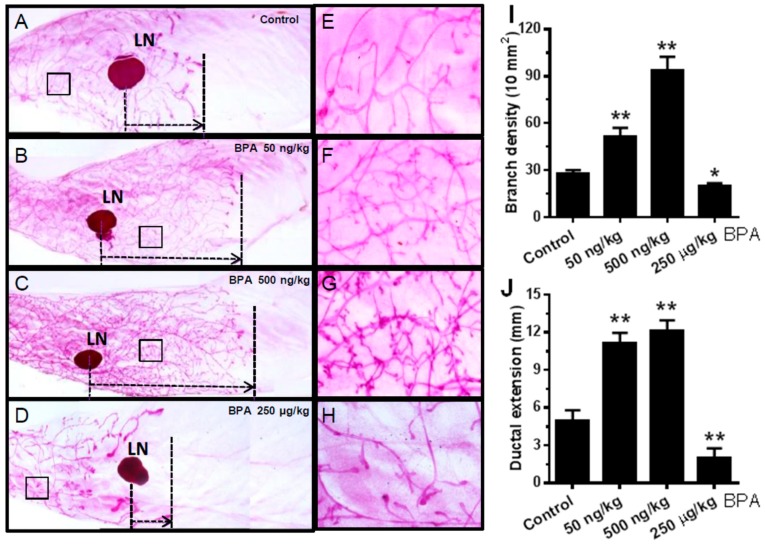
In utero exposure to BPA has a profound impact on mammary morphogenesis at PND 70. Mammary whole mounts were prepared from mice with in utero exposure to 0 (**A**,**E**), 50 ng/kg (**B**,**F**), 500 ng/kg (**C**,**G**), or 250 µg/kg (**D**,**H**) bodyweight of BPA daily between GD 11 and 19. The number of branch points per 10 mm^2^ and the duct extension beyond the lymph node of the mammary glands in different groups were quantified in panels (**I**,**J**), respectively. LN, lymph node. Five glands from each group were examined (* *p* < 0.05; ** *p* < 0.01).

To determine the effect of in utero exposure to BPA on mammary development beyond puberty, we examined the morphogenesis of young adult mice (PND 70) exposed to BPA in utero. As shown in Figure 3, mammary glands from mice exposed to low doses of BPA in utero displayed striking prolonged ductal extension and more complex lateral branching/alveolar structures relative to the control group. Interestingly, in utero exposure to the high dose of BPA impaired mammary development in a different way. Little ductal growth beyond the lymph node (a landmark of ductal extension) and fewer lateral branches suggest high dose BPA impedes and distorts mammary development. Altogether, these morphogenesis data indicate that in utero exposure to BPA-induced tumorigenesis, especially at low doses, is preceded with profound morphogenic changes in premalignant mammary tissues, which underscores the connection between reprogrammed mammary development and altered tumorigenic risks.

### 2.4. In Utero Exposure to BPA Induces Mammary Epithelial Cell Repopulation

**Figure 4:**
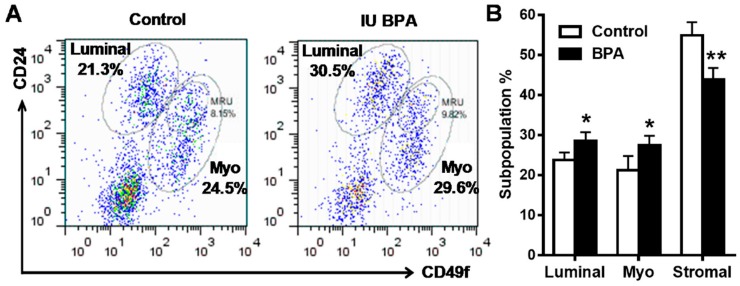
In utero exposure to low dose BPA affects mammary cell subpopulations in MMTV-erbB2 transgenic mice. Primary mammary epithelial cells of MMTV-erbB2 mice (PND 35) from control and low dose BPA (500 ng/kg) groups were stained with fluorescent CD24 and CD49f antibodies and analyzed using FACS. Representative plots of epithelial cell populations from mice with different treatments are shown with the percentage of luminal, basal/myoepithelial, and MRU/MaSC cells indicated (**A**). The average subpopulation percentage of luminal, basal/myoepithelial, and stromal cells (CD24^low^/CD49f^low^) from control and BPA-treated mice are quantified in (**B**) (* *p* < 0.05; ** *p* < 0.01).

Recent advances in cancer/mammary stem cell studies suggest that deregulation of mammary stem cell dynamics play a critical role in mammary tumor initiation [45]. Epithelial cell analysis with flow cytometry provide a powerful tool in the characterization and understanding of mammary hierarchy associated with altered developmental dynamics [46,47]. FACS analysis based on CD24 and CD49f/CD29 has been used to determine the relative composition of mammary epithelial cell subpopulations [46]. We therefore employed this approach to analyze mammary tissues of mice from control and low dose BPA (500 ng/kg) groups at PND 35. Using CD24 and CD49f cell markers, we were able to identify three main cell populations: luminal (CD24^high^/CD49f^mid^), basal/myoepithelial cells (CD24^mid^/CD49f^high^), and stromal cells (CD24^low^/CD49f^low^) [46] (Figure 4A). After analyzing the percentage of the cells in each subpopulation, we found that in utero BPA exposure significantly increased the luminal and myoepithelial cell populations as compared to the control (Figure 4B). Since cells in the luminal and basal/myoepithelial subpopulations contain luminal progenitor cells and mammary repopulating unit (MRU) cells (functional unit of mammary stem cells), respectively [46], an increase in both subpopulations suggests that in utero exposure to BPA-induced morphogenesis and tumor risk might be associated with enhanced mammary stem cell (MaSC) stemness. These data present a novel exploration of in utero BPA exposure on mammary epithelial cell reprogramming and uncover a fundamental change in epithelial cell dynamics that may increase the oncogenic potential of these cells.

### 2.5. In Utero Exposure to Low Dose BPA Induces Crosstalk between ER and EGFR/erbB2 Pathways

**Figure 5:**
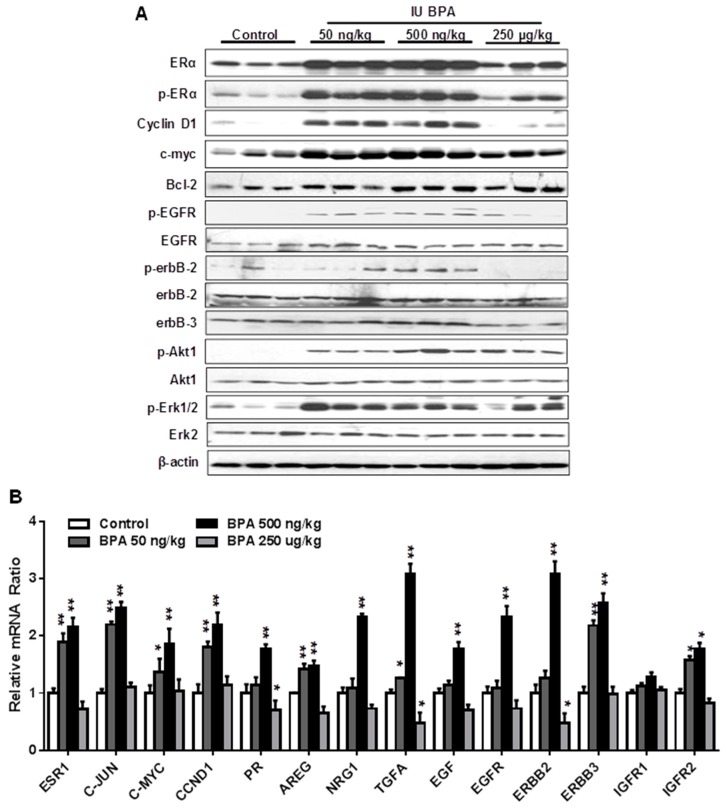
In utero exposure to low dose BPA induces concurrent activation of ER and EGFR/erbB2 pathways. (**A**) Western blot analysis of total and phosphorylated protein levels of indicated key regulators in ER and EGFR/erbB2 signaling pathways. Protein lysates were extracted from the mammary tissues of mice at PND 70 with different in utero BPA exposures, as detailed in the methods. (**B**) mRNA levels of genes involved in ER and RTK signaling in the mammary tissues of PND 70 mice with in utero exposure to indicated doses of BPA were quantified using real-time PCR. Each group was based on three samples from different mice with the same treatment (* *p* < 0.05; ** *p* < 0.01).

Previous studies reported that in utero exposure to BPA induces pro-estrogenic effect and upregulation of ER signaling in other models [25,41]. However, molecular changes of other pathways are understudied. To understand the mechanism of in utero exposure to BPA-associated tumor promotion in MMTV-erbB2 mice, we examined signaling in the ER and EGFR/erbB2 pathways in premalignant mammary tissues (PND 70) from mice with different in utero treatments. We found that in utero exposure to low doses of BPA, but to a lesser degree in the high dose group, induced significant increase in both total and phosphorylated/activated ERα (Figure 5A). Consistently, protein levels of Cyclin D1, c-myc, and Bcl-2, classical targets of ER pathway, were also upregulated. These results suggest that in utero exposure to BPA-induced deregulation of ER signaling is a fundamental mechanism that mediates phenotypic changes. We further examined the expression and activation of key markers in the EGFR/erbB2 pathway, including total- and phosphorylated- EGFR, erbB2, erbB3, Akt, and Erk1/2, in the PND 70 mammary tissues. We found that the activation/phosphorylation of the RTK signaling was in a pattern similar to the ER signaling, as indicated by marked increase in kinase activation in mammary tissues with in utero exposure to low doses, but not in high dose, of BPA (Figure 5A). As activation of Akt and Erk induces ERα phosphorylation/activation [48], the concomitant activation of both ER and EGFR/erbB2 pathways suggest a functional crosstalk between the two pathways. To understand the underlying mechanism, we examined the mRNA levels of key genes involved in ER signaling (ESR1, JUN, MYC, CCND1, PR) and RTK regulation (AREG, NRG1, EGF, EGFR, ERBB2, ERBB3, IGFIR1, IGFIR2) (Figure 5B). The results showed that most of the above genes were significantly upregulated in mice with in utero exposure to low doses of BPA. Since several genes involved in RTK signaling are also putative ER target genes, such as EGF, EGFR, IGFIR, AREG, and TGFα [48,49], upregulation of these genes by the pro-estrogenic effect associated with in utero exposure to BPA may serve as the mediators of ER-EGFR/erbB2 crosstalk. These data indicate that in utero exposure to low dose BPA induces concurrent functional activation of ER and EGFR/erbB2/Akt/Erk pathways, and suggests that enhanced ER-EGFR/erbB2 crosstalk in premalignant mammary tissues plays a critical role in tumorigenesis later in life.

### 2.6. In Utero Exposure to Low Dose BPA Also Induces ER-EGFR/erbB2 Crosstalk in FVB/N Mice

**Figure 6:**
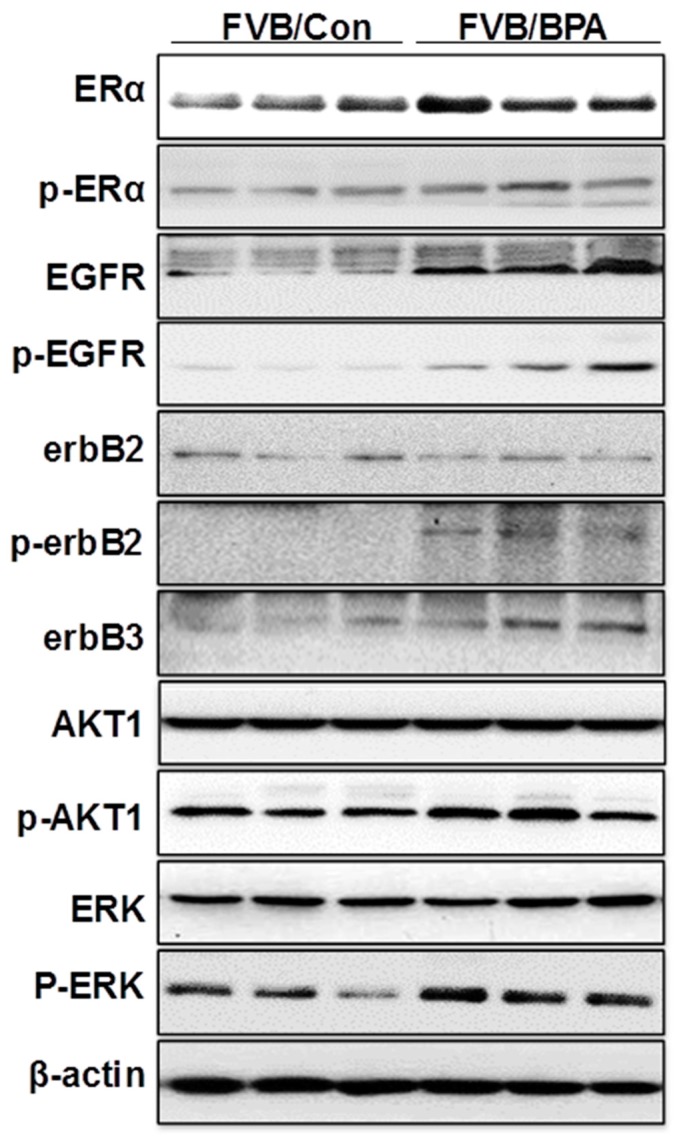
In utero exposure to low dose BPA induces ER-RTK crosstalk in mammary tissues of FVB/N mice. Pregnant FVB/N mice were treated with vehicle (FVB/Con) or 500 ng/kg BW of BPA (FVB/BP) via subcutaneous injection, daily between GD 11 and 19. The lysate was prepared from the mammary tissues of female offspring at PND 70, followed by Western blot analysis. Total and phosphorylated protein levels of indicated key regulators in ER and EGFR/erbB2 signaling pathways were examined.

Our data that in utero exposure to low dose BPA induces ER-EGFR/erbB2 crosstalk is an interesting finding that advances our understanding of the underlying mechanisms. However, since the MMTV-erbB2 transgenic mice have erbB2 overexpression in the mammary tissues, it is important to determine whether this mechanism is also involved in individuals without erbB2 overexpression. Therefore, we examined the effect of in utero exposure to low dose BPA on ER and RTK signaling in mammary tissues of FVB/N mice, which are the parental strain of the MMTV-erbB2 mice and do not overexpress erbB2. As shown in Figure 6, the protein levels of ERα, *p*-ERα, EGFR, *p*-EGFR, erbB3, *p*-AKT, and *p*-ERK1/2 were upregulated in the mammary tissues of FVB/N mice with in utero exposure to low dose (500 ng/kg BW) BPA. Concurrent upregulation/activation of key regulators of the ER and RTK pathways in the parental mice indicated that in utero exposure to low dose BPA-induced ER-EGFR/erbB2 crosstalk is an essential mechanism that mediates morphogenic and proliferative changes in this pathogenic process.

## 3. Discussion

In the present study, we investigated in utero exposure to BPA-associated mammary tumor development in MMTV-erbB2 transgenic mice. We found that prenatal exposure to low doses of BPA promoted mammary tumor development in this clinically relevant model, which was preceded with significant alterations in epithelial cell proliferation, morphogenesis, and signal transduction in ER and erbB2 pathways in premalignant mammary tissues. Results from this study advance our understanding of in utero exposure to BPA-induced susceptibility to mammary tumor development with mechanistic insight. Data from this study also establishes a model system for further mechanistic studies.

Increasing studies support the concept of developmental origins of health and disease (DOHaD), which emphasizes the impact of changes of pre- and peri-natal environment on adult diseases susceptibility [50]. Lessons from cancer risks associated with “DES daughters” have led to studies on breast/mammary tumor risk associated with in utero exposure to other hormonal disruptors [4]. Most previous studies on in utero exposure to BPA used carcinogen models [24,25,26,27], which have generated substantial proof-of-concept data. However, because the molecular responses of the carcinogenic model may be different from human breast carcinogenesis, mechanistic studies using these models are compromised. In contrast, MMTV-erbB2 transgenic mice have a defined genetic background and are a well-established clinically relevant model for erbB2-mediated carcinogenesis, which is also often used to test environmental factor-modulated mammary tumor risk [51,52,53]. The long tumor latency (average 36 weeks) of this model also allows us to assess even subtle effects of etiologic factors, such as in utero exposure to cancer-promoting substances [54]. Results from this model demonstrate that in utero exposure to BPA may interact with predispositions of deregulated RTK signaling to promote mammary tumor development. Hence, our studies were not only for additional proof of concept support but also for establishing a novel model system and looking for mechanistic insight, such as the mammary epithelial reprogramming.

In this study, we found that in utero exposure to various doses of BPA (0, 50, and 500 ng/kg BW) induced profound changes in mammary development and tumor risk. In particular, in utero BPA exposure to a dose of 500 ng/kg BPA, but not the high dose group, significantly promoted mammary tumor development in the erbB2 mice. In context with our preliminary studies using high doses and the reports from others [26,55], the effects of in utero exposure to BPA on mammary development and tumor risk are very sensitive to doses and exposure conditions. However, the effects were not in a simple linear pattern. Consistent with the nonmonotonic dose response observed in a study testing adult BPA exposures [51], we demonstrated that low doses, but not high doses, of BPA may induce more adverse effects. This is consistent with the growing concerns over low-dose BPA-associated cancer risk [56]. The boundary between “low” and “high” doses may be affected by route of administration and other conditions, which warrants further investigation. In previous studies, BPA exposures included drinking water, subcutaneous injection, gavage, and osmotic pump, which yielded various responses [5,20,51,57,58]. To have an accurate measurement of exposure conditions, we used subcutaneous injection of BPA in this study. BPA exposure equivalent to 500 ng/kg BW is in the range of environmentally relevant exposures in humans [59]. With the fundamental data from this transgenic model, future detailed dose-response analyses will be followed to evaluate the influence of different doses and routes of administration on serum levels of BPA to improve this model system. Previous studies reported that prenatal BPA exposure induced pro-estrogenic effects on pubertal mammary glands in CD-1 mice [60]. To understand the underlying mechanism of modified tumorigenesis, we used multiple approaches to characterize histopathological changes of mammary tissues with different in utero exposures at different ages (Figure 2, Figure 3, Figure 4 and Figure 5). We showed that in utero exposure to low doses of BPA induced earlier vaginal opening and disruption of estrous cycles. The proliferative mammary morphogenesis at puberty is reflected by increased TEB and BrdU incorporation (Figure 2). Importantly, prolonged ductal extension and increased branching density in PND 70 mammary tissues of low dose groups indicate persistent changes associated with in utero exposure to BPA (Figure 3). Conversely, in utero exposure to high dose (250 µg/kg) BPA induced impaired mammary development, as indicated by impeded ductal growth/development that may involve altered differentiation (Figure 2 and Figure 3), although tumor latency was not shortened. Integrating the tumorigenesis data (Figure 1), our results indicate that in utero exposure to low doses of BPA-induced accelerated tumorigenesis, and was preceded with altered pubertal glands and persistent proliferative morphogenesis.

It is now known that MaSCs are the driving force for mammary development [47]. Increasing studies also suggest that MaSCs are the targets of different carcinogenic factors and their deregulation may lead to the development of cancer stem cells and tumor initiation [45]. A recent study reported that pubertal exposure to BPA-induced MaSC alteration was associated with the development of neoplastic lesions [20]. Whether and how in utero exposure to BPA modifies MaSC dynamics is an interesting topic. Since it has been observed in several studies that in utero exposure to BPA induces increased TEB numbers in pubertal glands and TEBs are enriched with MaSCs [44], alterations of MaSCs was well speculated [61]. Data from our FACS analysis of mammary epithelial subpopulations, which showed increased luminal and basal subpopulations in pubertal glands with in utero exposure to low dose BPA (Figure 4), indicate altered mammary developmental/differentiation dynamics in these animals. Profound alterations in morphogenesis and developmental reprogramming underscore the significance of studies on MaSC deregulation in premalignant mammary tissues [62]. These data demonstrated in utero exposure to BPA-induced mammary epithelial cell reprogramming and laid a foundation for further characterization of alterations in MaSCs and progenitor cells.

Several studies on in utero exposure to hormonal disruptors, including BPA, have reported that these prenatal exposures induce pro-estrogenic effects and deregulation of estrogen signaling [25,41]. However, molecular changes beyond this have been rarely studied. Our data demonstrated that in utero exposure to low dose BPA induced concomitant upregulation of both ER and ErbB/RTK pathways in mammary tissues (Figure 5A), which was correlated with proliferative morphogenesis and the altered tumor risk, suggesting the critical role of ER-ErbB/RTK crosstalk in this process. Data from the qPCR analysis indicated that enhanced ER-ErbB/RTK crosstalk was accompanied by upregulation of EGF-like ligands, such as EGF, AREG, and TGFα, which are also ER target genes, which may function as the mediators of the crosstalk (Figure 5B). Based on our data, it is suggested that the pro-estrogenic activity induces the expression of ErbB ligands and receptors that lead to the activation of the RTK signaling. The resulted activation of Akt and Erk1/2 phosphorylates/activates ERα that further amplifies the crosstalk, which promotes mammary proliferation and tumor risk [48]. Of note, the ER-ErbB/RTK crosstalk is not limited in mammary tissues with erbB2 overexpression. Our data based on FVB/N mice, the parental strain of the MMTV-erbB2 mice, showed that in utero exposure to BPA also induced upregulation of ER and RTK signaling in mammary tissues (Figure 6), which might be mediated by the activation of EGFR, ErbB3, and their ligands. Taken together, our studies advance our understanding of in utero exposure to BPA-induced mammary development and tumor risk with mechanistic insights.

Overall, our study demonstrated that in utero exposure to low dose BPA promotes mammary tumor development in MMTV-erbB2 transgenic mice. Morphogenesis characterization revealed that the in utero exposure induced persistent proliferative glands with mammary epithelial cell repopulation. The underlying mechanisms involve upregulation of the ER-ErbB/RTK crosstalk and developmental reprogramming. The phenotypic changes identified in this study may serve as a reference for developing biomarkers for clinical evaluation. Our results highlight the significance of prenatal exposure to BPA in breast cancer risk later in life, which underscores the significance of prenatal factors in breast cancer etiology. Our model system provides a novel platform for further mechanistic studies.

## 4. Materials and Methods

### 4.1. Reagents

BPA was purchased from Sigma Company (St. Louis, MO, USA). Primary monoclonal antibody against erbB2 was obtained from Millipore Corporation (Temecula, CA, USA). Antibodies against ERα, phospho-ERα, EGFR, phospho-EGFR, Akt1, phospho-Akt, phospho-erbB2, and β-actin were obtained from Cell Signaling (Danvers, MA, USA). Antibodies against erbB3, ERβ, phospho-ERβ, Erk2, phospho-Erk1/2, Bcl-2, c-myc, and Cyclin D1 were purchased from Santa Cruz Biotechnology Inc. (Santa Cruz, CA, USA). Monoclonal mouse antibody against BrdU was obtained from Invitrogen (Camarillo, CA, USA). BrdU was purchased from Roche Applied Sciences (Indianapolis, IN, USA). Secondary antibodies included anti-mouse and anti-rabbit Horseradish peroxidase (HRP) from Thermo Scientific (Waltham, MA, USA).

### 4.2. Animals and Treatments

FVB/N-Tg(MMTV-neu) (MMTV-erbB2) transgenic mice and parental FVB/N mice were from Jackson Laboratory (Bar Harbor, ME, USA) and housed in the institutional animal facility. Animal care and experiments were conducted in accordance with the protocols approved by the Institutional Animal Care and Use Committee. All animals used in this study were fed estrogen-free AIN-93G semipurified diet (Bio-Serv., Flemington, NJ, USA) ad libitum.

Female and male mice were mated at 8 weeks of age. On GD 11, pregnant mice were randomly assigned to individual experimental groups. Each group had 30 mice and were injected subcutaneously with 50 µL of either 50 ng BPA/kg BW (ng/kg BW), 500 ng/kg BW, 250 µg/kg BW, or vehicle corn oil. BPA was dissolved in 0.1% ethanol/corn oil and administered daily to pregnant mice during GD 11–19. All female offspring were weaned on postnatal day (PND) 21 and continued on the same estrogen-free diet.

Vaginal opening of the female offspring was examined starting at PND 20. Vaginal smears of mice were examined daily between PND 42 to 60. The estrous cycle phases were characterized with Giemsa staining. Beginning at 20 weeks of age, mammary tumor development was observed twice a week for all female offspring until the end point of the experiments, i.e., when the tumors reached 1.5 cm in diameter. After palpable tumors were detected, tumor sizes were measured with calipers twice a week. Tumor latency and the percentages of tumor-free mice were calculated at the end of the experiment. Mammary tissues at different ending points were collected for mammary gland whole mount, FACS, protein, and DNA/RNA analyses.

### 4.3. Whole Mount Analysis

The inguinal mammary glands collected from each mouse at indicated time points were fixed in Carnoy’s solution overnight, followed by re-hydration and carmine alum staining [52]. The whole mounts were captured using Nikon Elements Imaging System (Nikon Instruments, Inc., Melville, NY, USA). The number of TEBs was counted for mice at PND 35. Ductal extension beyond the lymph node (LN) was measured and the number of branches was counted for six fields per gland of mice at PND 70. Average branching was calculated to determine ductal branching density per 10 mm^2^.

### 4.4. 5-Bromo-2′-Deoxyuridine (BrdU) Incorporation and Immunohistochemistry (IHC)

For BrdU incorporation analysis, mice were injected via intraperitoneal injection with 0.6 mg BrdU in 200 µL ddH_2_O two hours prior to sacrifice. Appropriate mammary tissues were collected and fixed for BrdU detection. Formalin-fixed paraffin-embedded sections were processed as previously described [63], followed by DNA denaturation with 2N HCl at 37 °C for 30 min for BrdU treated samples. Conditions for blocking, antibody incubation, washing, staining, and color development using the Vectastain ABC kit (Vector Laboratories, Burlingame, CA, USA) were the same as in previous reports [63]. Under a Nikon Eclipse microscope, the number of mammary epithelial cells showing BrdU-stained nuclei was recorded to calculate the percentage of proliferating cells in each group.

### 4.5. FACS Analysis of Mammary Epithelial Cell Subpopulations with CD24 and CD49f

Mammary gland tissue was homogenized with a tissue chopper (Mickle Laboratory Engineering, United Kingdom), immediately followed by digestion for 2 h at 37 °C using collagenase and hyaluronidase (Sigma, St. Louis, MO, USA). Organoids that developed were subsequently digested again with 0.25% Trypsin-EDTA (Sigma) and Dispase (Sigma)/DNase I (Sigma). To get the final single cell suspension, the cells were strained through a 40 µm mesh filter. The single cell suspension of isolated primary MECs was then used for flow cytometry analysis below [64,65]. For flow cytometry analysis, the primary MECs were stained with fluorescent antibodies against lineage and cell surface markers, according to standard procedures [65]. Briefly, cells were stained with 7-amino-actinomycin D to exclude non-viable cells; purified anti-CD16/CD32 (BD Biosciences, San Jose, CA, USA) to block Fc receptors; PE-conjugated anti-CD31, anti-TER-119 and anti-CD45 (BioLegend, San Diego, CA, USA) to exclude endothelial, erythroid and leukocyte cells, respectively; and biotin–streptavidin–APC anti-CD24 (BD Biosciences) and FITC-conjugated anti-CD49f (BD Biosciences). FlowJo analysis software was used for gating and quantification of the individual cell populations.

### 4.6. Western Blotting

Mammary gland tissue was snap-frozen in liquid nitrogen after collection. Tissues were homogenized in NP-40 lysis buffer supplemented with protease inhibitors, followed by centrifugation at 4 °C. Protein concentration in the supernatant was measured using the BCA Protein Assay kit (Thermo Scientific). Fifty (50) µg from each sample were separated with a 10–12% SDS-PAGE gel, and were subsequently transferred onto nitrocellulose membranes. After blocking with 5% dry milk in TBS-T, the membranes were then incubated with indicated primary antibodies (diluted 1:1000–1:2000 in 5% BSA in TBS-T) at 4 °C overnight and washed with TBS-T prior to the incubation of secondary antibodies for 1 h at room temperature. Specific bands were visualized using Fluorchem E system (Cell Biosciences; San Jose, CA, USA) following the incubation with enhanced chemiluminescence reagents (Thermo Scientific) [63].

### 4.7. RNA Isolation, Reverse Transcription, and Real-Time PCR

RNA was extracted from mammary gland tissues using RNeasy Protect Mini Kit (Qiagen, Germantown, MD, USA) according to the manufacturer’s instructions. Reverse transcription was completed using iScript cDNA Synthesis Kit (BioRad, Hercules, CA, USA) with 1 μg of RNA template. Real-time (RT) PCR was performed using SsoFast EvaGreen Supermix Kit (20 µL reaction volume containing 50 ng of cDNA, 0.4 µM forward and reverse primers, and 10 µL SsoFast EvaGreen Supermix; BioRad). The primers used are shown in Supplementary Table S1. Cycling steps are as follows: denaturation at 95 °C for 30 s, followed by 39 amplification cycles at 95 °C for 5 s, and 60 °C for 5 s. The relative fold changes of mRNA expression were quantified by normalizing the cycle threshold (Ct) value of the experimental gene to the mean Ct value of the control β-actin gene.

### 4.8. Statistical Analysis

The Kaplan–Meier method was used to calculate the percentages of tumor-free mice by the endpoint of the experiment and the average latency of mammary tumors in each group. The significance of tumor latency was analyzed with a log-rank test. The significant differences of vaginal opening days were analyzed using non-parametric test, and estrous cycles, TEB, ductal extension, and branch density among the groups were determined by Student’s *t*-test. A *p*-value of ≤ 0.05 was considered statistically significant for all analyses.

## Figures and Tables

**Table 1:** The effect of in utero exposure to BPA on vaginal opening dates.

Group	Day 26	Day 27	Day 28	Day 29	Day 30	Day 31	Vaginal Opening Day (Mean ± S.E.)	*p*-Value
**Control**			1	4	8	2	29.7 ± 0.2	
**BPA 50 ng/kg**	4	2	4	2	3		27.9 ± 0.4	<0.01
**BPA 500 ng/kg**		3	5	5	2		28.4 ± 0.3	<0.01
**BPA 250 µg/kg**		2	3	4	5	1	29.0 ± 0.3	

Table 1 The effect of in utero exposure to BPA on vaginal opening dates in MMTV-erbB-2 mice. Vaginal opening of the female offspring with different in utero treatments were examined daily between postnatal day 20 and 36. For in utero treatment, mothers of corresponding groups were exposed to BPA via subcutaneous injection of corn oil containing 0 (vehicle only), 50 ng/kg, 500 ng/kg, or 250 µg/kg BPA daily between gestational day 11 and 19 (*n* = 15), respectively. The data was analyzed with non-parametric test.

**Table 2:** In utero exposure to low dose BPA interrupts the estrous cycle.

Group	Days in Estrus Phase (Mean ± S.E.)	*p*-Value
**Control**	5.2 ± 0.4	
**BPA 50 ng/kg**	7.4 ± 1.1	<0.01
**BPA 500 ng/kg**	7.2 ± 0.8	<0.01
**BPA 250 µg/kg**	5.0 ± 0.7	

Table 2 The effect of in utero exposure to BPA on estrous cycle in MMTV-erbB-2 mice. Pregnant mothers were treated with BPA as detailed in Table 1. Vaginal smears of the female offspring with in utero exposure to BPA were examined daily between PND 42-60. The estrous cycle phases were characterized with Giemsa staining. The total number of days in the estrus phase during the two-week period (14 days) was analyzed among the different groups with Student’s *t*-test.

## Supplementary Materials

The following are available online at https://www.mdpi.com/1422-0067/21/9/3095/s1, Supplementary Table S1. Primer sequences used for RT-PCR.

## Abbreviations

BPA	Bisphenol A
DES	diethylstilbestrol
EDCs	Endocrine disrupting compounds
ER	estrogen receptor
GD	gestational day
TICs	tumor initiation cells
TEBs	terminal end buds
RTK	receptor tyrosine kinase
PND	postnatal day
MRU	mammary repopulating unit
MaSCs	mammary stem cells
