# Supplementary material for: In Utero Exposure to Bisphenol a Promotes Mammary Tumor Risk in MMTV-Erbb2 Transgenic Mice Through the Induction of ER-erbB2 Crosstalk
Source: Int J Mol Sci. 2020 Apr 28;21(9):3095. doi: 10.3390/ijms21093095 (PMC7247154; doi:10.3390/ijms21093095)
Supplement: Supplementary file 1 [file ijms-21-03095-s001.pdf]

Supplementary Table S1. Primer sequences used for RT-PCR.

Primer Name	Sequence
ESR1	F: 5'-TCTCTGGAAGAGAAGGACCACATC-3'<br>R: 5'-TGCAGAGTCAGGCCAGCTTT-3'
JUN	F: 5'-AAAACCTTGAAAGCGCAAAA-3'<br>R: 5'-GTTTGCAACTGCTGCGTTAG-3'
MYC	F: 5'-TGAGCCCCTAGTGCTGCA-3'<br>R: 5'-AGCCCGACTCCGACCTCTT-3'
CCND1	F: 5'-GGGCACCTGGATTGTTCT-3'<br>R: 5'-CACCGGAGACTCAGAGCA-3'
PR	F: 5'-CACAGCGCTTCTACCAACTCACAA-3'<br>R: 5'-TTGGGCAACTGGGCAGCAATAA-3'
AREG	F: 5'-ACCTGGAGGTGGTGACATGCA-3'<br>R: 5'-TGCCGATGCCAATAGCTGCGA-3'
NRG1	F: 5'-TTCATCACAACCCTGCACAT-3'<br>R: 5'-GAACTTGGGTTGCTGTCCAT-3'
TGFA	F: 5'-GTGAGTGGTGCCGTGCCCTC-3'<br>R: 5'-CGAGCCCAGCAGGCAGCTTT-3'
EGF	F: 5'-TTTTGCCTCAGAAGGAGTGC-3'<br>R: 5'-GGCCACACTTGGCAGTATATC-3'
EGFR	F: 5'-TGGTAAGTCAGGGGCAAGTC-3'<br>R: 5'-ACATGGCACTTCCTGGTGAT-3'
ERBB2	F: 5'-CAGCCCCAGAGGATTACAGA-3'<br>R: 5'-TCAGTCCTAGTGGGGTGTCC-3'
ERBB3	F: 5'-GAGCTTCCAGACTCCGTTTG-3'<br>R: 5'-AAATGGCCTGCAGCTTACAC-3'
IGFIR1	F: 5'-TCTTGGATGCGGTGTCCAATAAC-3'<br>R: 5'-GCAGCACTCATTGTTCTCGTTGC-3'
IGFIR2	F: 5'-TGCACACTCTTCTTCTCCTGGCA-3'<br>R: 5'-GCAGATGTTGATATAGAAGTCAGG-3'
ACTB	F: 5'-TGGAATCCTGTGGCATCCATGAAAC-3'<br>R: 5'-TAAAACGCAGCTCAGTAACAGTCCC-3'
